# Re-highlighting the action of PPARγ in treating metabolic diseases

**DOI:** 10.12688/f1000research.14136.1

**Published:** 2018-07-24

**Authors:** Sung Hee Choi, Sung Soo Chung, Kyong Soo Park

**Affiliations:** 1 Department of Internal Medicine, Seoul National University College of Medicine, Seoul, South Korea; 2 Department of Internal Medicine, Seoul National University Bundang Hospital, Seongnam, South Korea; 3 Biomedical Research Institute, Seoul National University Hospital, Seoul, South Korea; 4 Department of Molecular Medicine and Biopharmaceutical Sciences, Graduate School of Convergence Science and Technology, Seoul National University, Seoul, South Korea

**Keywords:** PPARgamma, post-translational modification, metabolic disease

## Abstract

Peroxisome proliferator-activated receptor γ (PPARγ) is a member of the nuclear receptor family and plays an important role in adipocyte differentiation, glucose homeostasis, and insulin sensitivity. Thiazolidinediones (TZDs), synthetic ligands of PPARγ, have been used for the treatment of diabetes mellitus for two decades. TZDs were expected to be amazing drugs not only for type 2 diabetes but also for metabolic syndrome and atherosclerotic vascular disease because they can reduce both insulin resistance and inflammation in experimental studies. However, serious unwanted effects pushed TZDs back to an optional second-tier drug for type 2 diabetes. Nevertheless, PPARγ is still one of the most important targets for the treatment of insulin resistance and diabetes mellitus, and novel strategies to modulate PPARγ activity to enhance its beneficial effects and reduce unwanted adverse effects are anticipated. Recent studies showed that post-translational modification (PTM) of PPARγ regulates PPARγ activity or stability and may be a novel way to optimize PPARγ activity with reduced adverse effects. In this review, we will focus on recent advances in PTM of PPARγ and the mechanisms regulating PPARγ function as well as in the development of PPARγ modulators or agonists.

## Introduction

Insulin resistance is the key pathophysiologic abnormality of many metabolic diseases such as type 2 diabetes mellitus, obesity, dyslipidemia, and cardiovascular diseases ^1^. Therefore, reducing insulin resistance is the most important strategy for improving metabolic deterioration. Thiazolidinediones (TZDs), peroxisome proliferator-activated receptor γ (PPARγ) agonists, have shown many beneficial effects not only by enhancing insulin sensitivity but also by demonstrating anti-inflammatory and antioxidant properties, whose actions are related to anti-atherosclerosis ^2, 3^. Thus, TZDs were considered a magic bullet for the treatment of type 2 diabetes and atherosclerosis. Indeed, TZDs demonstrated a preventive role for recurrent ischemic stroke in several clinical trials ^4^ and for restenosis after percutaneous coronary intervention (PCI) ^5– 7^. However, TZDs increased the risk of peripheral edema, bone loss, and congestive heart failure ^8– 10^. A meta-analysis of clinical trials showed that rosiglitazone significantly increased the risk of myocardial infarction ^11^. Although later studies revealed that rosiglitazone did not increase the risk of heart attack and the US Food and Drug Administration (FDA) removed the warning labels from rosiglitazone-containing drugs regarding the issue of increasing heart attack in 2013, rosiglitazone’s cardiovascular safety issue alongside the above-mentioned adverse effects still lead to many physicians hesitating to prescribe TZDs in their clinical practice. Nevertheless, PPARγ is still one of the most important targets for the treatment of insulin resistance and type 2 diabetes, and novel strategies to modulate PPARγ activity to enhance its beneficial effects and reduce unwanted adverse effects are strongly anticipated. Recent studies showed that post-translational modification (PTM) of PPARγ regulates PPARγ activity or stability and may be a novel way to optimize PPARγ activity with reduced adverse effects. In addition, selective PPARγ modulators (sPPAR *γ*Ms), dual or pan PPAR agonists, have been developed and tested for their metabolic effects in animal studies and in some clinical trials.

## PPARγ, a therapeutic target for insulin resistance ( Figure 1)

PPARγ is a master regulator of adipocyte differentiation. It is also involved in glucose homeostasis and insulin sensitivity. The expression of PPARγ is most abundant in adipose tissue ^12^. Evidence has shown that the primary target of TZDs is adipose tissue, where it increases the expression of Glut4 and CAP ^13^, and an animal model lacking PPARγ in adipose tissue had a significantly lower response to TZDs ^14, 15^. TZDs inhibit the expression of TNF-α, IL-6, and resistin in adipose tissue, which promote insulin resistance and chronic inflammation ^16, 17^, while TZDs increased the production of adiponectin and fibroblast growth factor 21 (FGF21), which enhance fatty acid oxidation and insulin sensitivity ^18, 19^. TZDs increase lipogenesis by aP2, LPL, CD36, fatty acid transport protein, PEPCK, and the glycerol transporter aquaporin 7 ^2^ and make adipose tissue store more lipid, while TZDs remove lipid accumulation in other tissues such as muscle and liver ^20^.

**Figure 1.**
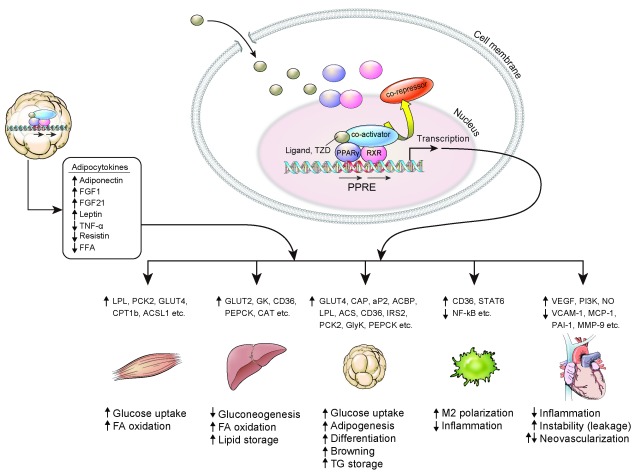
Effect of PPARγ activation on various tissues. ACSL1, acyl-CoA synthetase long chain family member 1; CD36, cluster of differentiation 36; CPT1b, carnitine palmitoyltransferase 1B; FA, fatty acid; FFA, free fatty acid; FGF, fibroblast growth factor; GK, glucokinase; GLUT, glucose transporter; GlyK, glycerol kinase; IRS2, insulin receptor substrate 1; LPL, lipoprotein lipase; NF-κB, nuclear factor kappa-light-chain-enhancer of activated B cells; MCP-1, monocyte chemoattractant protein 1; MMP-9, matrix metalloproteinase 9; NO, nitric oxide; PAI-1, plasminogen activator inhibitor type 1; PCK2, peroxisome proliferator-activated receptor gamma 2 binding site; PEPCK, phosphoenolpyruvate carboxykinase; PI3K, phosphoinositide 3-kinase; PPARγ, peroxisome proliferator-activated receptor γ; PPRE, peroxisome proliferator-activated receptor response element; RXR, retinoid X receptor; STAT6, signal transducer and activator of transcription 6; TG, triglyceride; TNF-α, tumor necrosis factor α; TZD, thiazolidinedione; VCAM-1, vascular cell adhesion molecule-1; VEGF, vascular endothelial growth factor.

From these studies, it seems that improvement of insulin sensitivity in liver and muscle might be secondary to the effects of TZDs in adipose tissue. However, there is also evidence showing that TZDs have an insulin-sensitizing effect on other peripheral organs. It has been demonstrated that ablation of liver PPARγ in mice reduced hepatic steatosis but worsened hyperlipidemia, triglyceride clearance, and muscle insulin resistance ^21^. The expression of PPARγ in skeletal muscle is relatively low compared to adipose tissue, and the physiological significance of PPARγ in skeletal muscle has been shown to work indirectly in previous studies ^22^. However, selective activation of PPARγ in skeletal muscle showed significant protection from high-fat diet-induced insulin resistance and associated changes in muscle phenotype, such as decreasing the quantity of lipid in myocytes and increasing the number of oxidative muscle fiber types ^23^. It suggests that the activation of PPARγ can act directly on muscle tissue to improve insulin sensitivity. Macrophage PPARγ is also implicated in anti-inflammation and lipid metabolism ^24^, and mice lacking macrophage PPARγ are more prone to whole-body insulin resistance ^25, 26^.

## PPARγ agonists and their effects on the vascular system: friend or foe?

PPARγ is expressed in the endothelium and vascular smooth muscle in the blood vessel wall ^27, 28^. Despite controversial cardiovascular effects of TZDs in humans, most experimental studies showed beneficial effects on vascular systems. TZDs inhibit the proliferation and migration of vascular smooth muscle cells (VSMCs), with potential favorable effects on atherosclerosis ^29, 30^. Smooth muscle-specific dominant-negative PPARγ transgenic mice showed a loss of nitric oxide responsiveness and high contractility ^31^, which resulted in systolic hypertension. In humans, dominant-negative mutations of PPARγ are associated with early hypertension and insulin resistance ^32^. Activation of PPARγ inhibits CCAAT/enhancer-binding protein-δ (C/EBPδ), which is a well-known mediator of the proinflammatory response in vascular cells ^33^.

TZDs also reduce activation and inflammation in endothelial cells by suppressing the expression of inflammation-associated genes ^34– 37^. On the other hand, TZDs induce vascular endothelial growth factor (VEGF) in endothelial cells and increase endothelial cell proliferation and migration by the Akt-dependent pathway ^38– 40^. In recent data, rosiglitazone significantly increased endothelial cell migration and vascular leakage in an animal study with increased VEGF expression and suppressed tight junction proteins, which caused instability of the endothelial membrane ^41^. This result could be related to vascular permeability, peripheral edema, and congestive heart failure associated with the use of TZDs, contrary to their beneficial effect on vascular cells. We still need more concrete evidence to understand the role of TZDs in the whole vascular system under various conditions.

## Regulation of PPARγ by PTMs to reduce the side effects of TZDs

**Figure 2.**
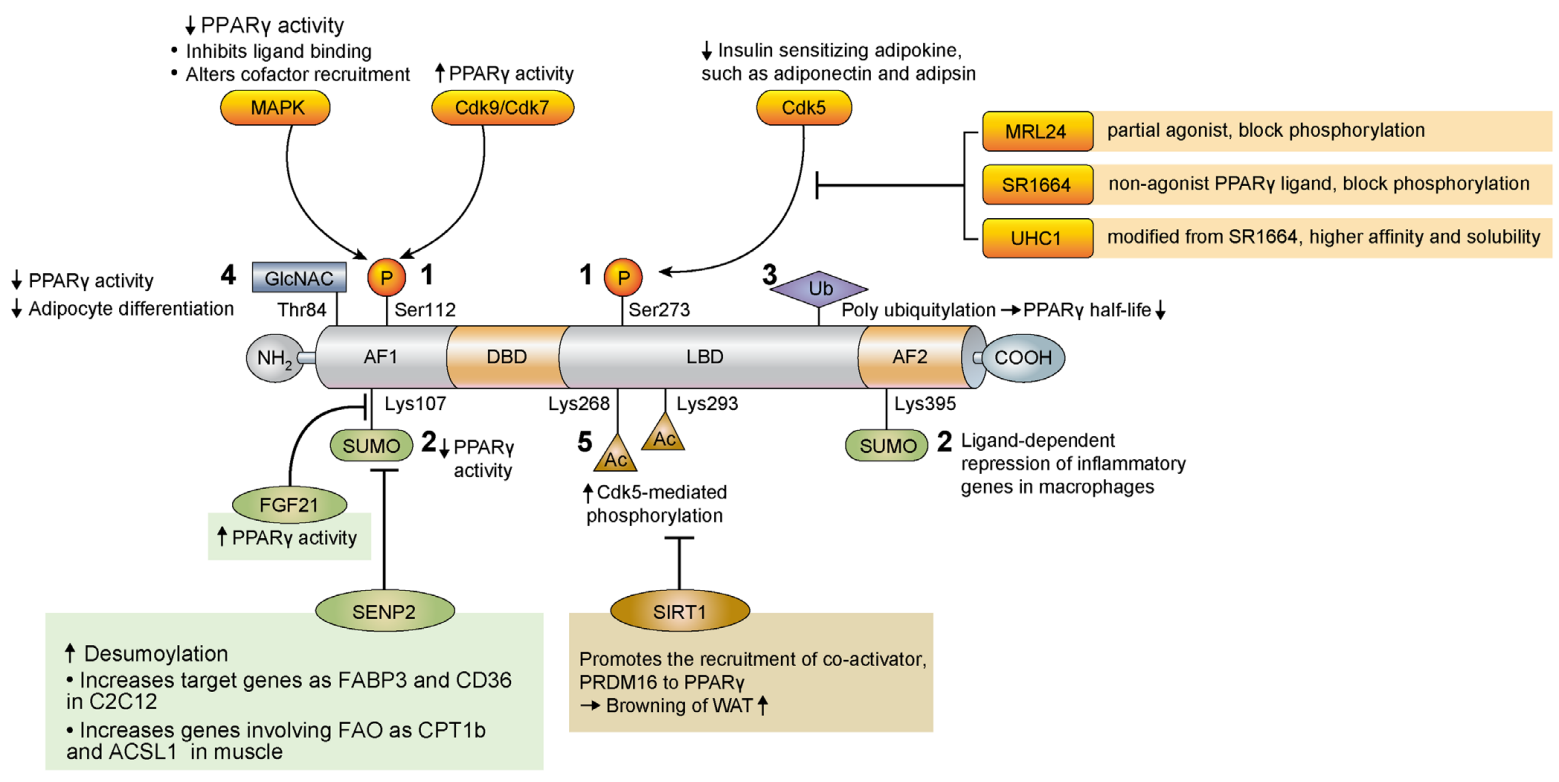
Regulation of PPARγ by post translational modification. Ac, acetyl; ACSL1, acyl-CoA synthetase long chain family member 1; AF1, activation function 1; AF2, activation function 2; CD36, cluster of differentiation 36; Cdk, cyclin-dependent kinase; CPT1b, carnitine palmitoyltransferase 1B; DBD, DNA-binding domain; FABP3, fatty-acid-binding protein 3, muscle and heart; FAO, fatty acid oxidation; FGF, fibroblast growth factor; GlcNAC, N-acetylglucosamine; LBD, ligand-binding domain; Lys, lysine; MAPK, mitogen-activated protein kinase; P, phosphate; PPARγ, peroxisome proliferator-activated receptor γ; PRDM16, PR domain containing 16; SENP2, small ubiquitin-like modifier-specific protease 2; Ser, serine; SIRT1, sirtuin 1; SUMO, small ubiquitin-like modifier; Thr, threonine; Ub, ubiquitin; WAT, white adipose tissue.

The PTM of PPARγ involves several pathways, including phosphorylation, SUMOylation, ubiquitination, β-O-linked N-acetylglucosamine modification (O-GlcNAcylation), and acetylation. These PTMs are known to regulate both PPARγ expression and its transcriptional activity ^42^ and have been recently suggested as a good modality for reducing the side effects of PPARγ activation by TZDs ^43^ ( Figure 2).

### Phosphorylation

Phosphorylation at serine 112 (S112) in the N-terminal AF-1 domain was first identified, and various studies revealed that net results of PPARγ phosphorylation may inhibit or stimulate its transcriptional activity depending on the cellular contexts and kinases involved ^44– 48^. Phosphorylation at S273 in the ligand-binding domain is mediated by cyclin-dependent kinase 5 (Cdk5), which is activated by pro-inflammatory stimuli and free fatty acids ^49^. S273 phosphorylation affects the expression of insulin-sensitizing adipokines such as adiponectin and adipsin but not those affecting adipogenesis. PPARγ partial agonist MRL24 specifically blocks the phosphorylation of PPARγ at S273 and has higher anti-diabetic activity and fewer side effects than does rosiglitazone ^49^. SR1664 and similar non-agonist PPARγ ligands were also developed for blocking cdk5-mediated phosphorylation and showed improved insulin sensitivity in high-fat diet-fed mice without causing side effects such as fluid retention and weight gain ^43, 50^. More recently, it has been reported that phosphorylation at S273 is also facilitated by MEK/ERK, and inhibition of MEK and ERK improves insulin resistance, suggesting that MEK and ERK inhibitors can be therapeutic targets for diabetes through the modulation of PPARγ function ^51^.

### SUMOylation

Small ubiquitin-like modifier (SUMO) modification is a reversible process and may affect protein stability, transcriptional activity, and protein–protein interaction. PPARγ is known as a target of SUMOylation. Lysine 107 (K107) of PPARγ2 is the major SUMOylation site, and deSUMOylation of this site increases the transcriptional activity of PPARγ ^52^. The K107R mutant form of PPARγ stimulates adipogenesis and suppresses neointimal formation after balloon injury more effectively than does the PPARγ wild-type form ^53, 54^. SUMOylation at K107 of PPARγ may be linked to S112 phosphorylation ^53^. PPARγ SUMOylation at K107 is markedly increased in FGF21-knockout mice, suggesting that FGF21 regulates PPARγ SUMOylation by an unknown mechanism ^19^. SUMOylation of PPARγ at K395 (K365 of PPARγ1) is stimulated by PPARγ agonists, and this modification inhibits the transcription of inflammatory response genes, such as *iNOS*, through recruiting transcriptional repressors to the NFkB complex in macrophages ^55^.

SUMO-specific protease 2 (SENP2) is the major deSUMOylation enzyme of PPARγ ^56^. Overexpression of SENP2 in C2C12 cells effectively induces PPARγ target genes such as *Fabp3* and *Cd36* but not *Adrp*; thus, SENP2 can induce the expression of PPARγ target genes in a selective manner ^56^. SENP2 deSUMOylates PPARγ and PPARδ and activates genes involved in fatty acid oxidation such as *Cpt1b* and *Acsl1*, which results in an increase of fatty acid oxidation in muscle. Interestingly, palmitate increases SENP2 expression via the TLR4-MyD88-NFkB pathway. These results suggest that SENP2 is an important regulator of fatty acid metabolism in skeletal muscle ^57^.

### Ubiquitination

Ubiquitination is the covalent attachment of ubiquitin, a 76-amino-acid peptide, to lysine residues in the substrate protein. PPARγ has a short half-life and is degraded by the polyubiquitin-proteasome pathway ^58^. Inhibition of proteasome activity by proteasome inhibitors increases PPARγ stability, suggesting ubiquitin modification of PPARγ is an important determinant of PPARγ activity ^58^. Several ubiquitin ligases, such as FBOX9 and Cul4B, and an ubiquitin-specific protease (HAUSP) targeting PPARγ have been identified, and an increase in PPARγ stability generally promotes PPARγ activity and adipogenesis ^59– 61^. Interestingly, PPARγ agonists, TZDs, stimulate the ubiquitination of PPARγ, which can be mediated by an ubiquitin ligase, Siah1 ^58, 62^. Therefore, PPARγ ubiquitination may be differently regulated by several ubiquitin E3 ligases or proteases upon various conditions.

### O-GlcNAcylation

O-GlcNAcylation is the post-translational cycling of a single β-O-linked N-acetylglucosamine (O-GlcNAc) on the hydroxyl groups of serine or threonine residues of target proteins. A major O-GlcNAc site in PPARγ is T84 in the AF-1 domain in PPARγ2, and increased O-GlcNAcylation reduces its transcriptional activity and adipocyte differentiation ^63^.

### Acetylation

Deacetylation at K268 and K293 by the NAD-dependent deacetylase sirtuin 1 (SIRT1) is necessary for the interaction of PPARγ with PRDM16, a transcriptional co-activator for the browning of WAT ^64^. Therefore, SIRT1-dependent PPARγ deacetylation selectively regulates PPARγ activity.

## Other PPARγ modulators and agonists

Considering that patients with insulin resistance show many conjugated metabolic problems such as atherosclerosis, obesity, fatty liver, etc., there have been many efforts to develop sPPAR *γ*Ms, dual or pan PPAR agonists, with potent efficacy but less-deleterious side effects.

sPPAR *γ*Ms bind to the ligand-binding domain of PPARγ in many ways, which leads to different receptor conformations and cofactor functions ^65^. INT131, a potent non-TZD sPPARγM now in clinical trials, showed excellent glucose lowering with significantly less weight gain, edema with fluid retention, and cardiomegaly than do current TZDs ^66^. Balaglitazone also showed positive effects in the treatment of patients with type 2 diabetes compared to placebo and pioglitazone (phase III clinical study, n = 409), better glycemic control, and less edema compared to pioglitazone 45 mg ^67^. CMHX008 was also tested for its effects in *in vitro* and *in vivo* models, showing excellent results by far ^68^.

PPARα/γ dual activation has been the focus of new targets from many pharmaceutical companies, and several clinical trials have been performed in potential treatments such as muraglitazar, tesaglitazar, and aleglitazar. However, owing to unpredictable side effects, the studies were all stopped for further development. Unfortunately, muraglitazar increased cardiovascular events, tesaglitazar increased renal toxicity, and aleglitazar showed bone fractures, heart failure, and gastrointestinal side effects ^69– 71^. Recently, (E)-N-(4-(3-(5-bromo-4-hydroxy-2-methoxyphenyl)acryloyl)phenyl)-4- *tert*-butylbenzamide (SN158) showed anti-diabetic effects through PPARα/γ dual activation. SN158 increased adipogenic differentiation of 3T3-L1 preadipocytes, enhanced fatty acid oxidation in hepatocytes, and increased glucose uptake in myotubes. It lowered plasma glucose and lipid levels in ob/ob mice without severe weight gain. Thus, it represents another candidate PPARα/γ agonist to enhance many metabolic profiles in obesity-related diseases ^72^.

There are some natural products which activate PPARγ and PPARα simultaneously or activate the PPARγ dimer partner retinoid X receptor. Compared to full TZDs, these natural products usually show fewer side effects and comparable anti-diabetic effects ^73^. Honokiol, amorfrutin 1, amorfrutin B, amorphastilbol, genistein, biochanin A, sargaquinoic acid, sargahydroquinoic acid, resveratrol, etc. were tested for their efficacy in *in vitro* and *in vivo* studies ^73^.

The development of PPARα/γ/δ pan agonists as anti-diabetic, anti-obesity, or hypolipidemic drugs is still actively ongoing ^74, 75^. For example, IVA 337 is a potent and well-balanced pan PPAR agonist which showed promising results *in vitro* and *in vivo* and is expected to be used to treat patients with metabolic syndrome and non-alcoholic steatohepatitis ^76^.

## Conclusion

PPARγ is still one of the most important targets for the treatment of insulin resistance and diabetes mellitus, even though current use of TZDs in clinical practice is limited because of undesirable adverse effects. Thus, novel strategies to modulate PPARγ activity to enhance its beneficial effects and reduce unwanted side effects have been strongly anticipated. Recent advances in understanding how PTM of PPARγ modulates PPARγ activity provide novel ways to optimize PPARγ activity with reduced adverse effects. In addition, selective PPARγ modulators, dual or pan PPAR agonists, have been developed and tested for their metabolic effects in animal studies and in some clinical trials.

We hope safer PPARγ agonists or modulators with excellent efficacy and fewer adverse effects will be available for treating metabolic diseases and insulin resistance in the near future.
